# Partnered Intimate Activities in Early Adolescence—Findings From the UK Millennium Cohort Study

**DOI:** 10.1016/j.jadohealth.2019.04.028

**Published:** 2019-06-21

**Authors:** Yvonne Kelly, Afshin Zilanawala, Clare Tanton, Ruth Lewis, Catherine H. Mercer

**Affiliations:** a Research Department of Epidemiology and Public Health, University College London, London, United Kingdom; b College of Public Health and Human Sciences, Oregon State University, Corvallis, Oregon; c Faculty of Public Health and Policy, London School of Hygiene and Tropical Medicine, London, United Kingdom; d MRC/CSO Social and Public Health Sciences Unit, Institute of Health and Wellbeing, University of Glasgow, Glasgow, United Kingdom; e Institute for Global Health, University College London, London, United Kingdom

**Keywords:** Partnered intimate activities, Social relationships, Parental monitoring and supervision, Health behaviors, Psychosocial well-being, Millennium Cohort Study

## Abstract

**Purpose:**

Little is known about potential influences on emerging partnered intimate behaviors in early adolescence. We investigate (1) the prevalence of partnered intimate activities and (2) associations with social relationships, parental monitoring and supervision, health behaviors, and psychosocial well-being.

**Methods:**

We used population-based data from the UK’s Millennium Cohort Study on 11,079 participants aged 14 years. Partnered intimate activities were grouped into three categories: “light” (handholding, kissing, and cuddling); “moderate” (touching and fondling under clothes); and “heavy” (oral sex and sexual intercourse). Multinomial logistic regression models were used.

**Results:**

Thirty percent of study participants reported not engaging in partnered intimate activity. Fifty-eight percent reported “light,” 7.5 percent “moderate,” and 3.2 percent “heavy” activity. Associated with increased likelihood (adjusted relative risk ratios [RRRs]) of intimate activities were confiding worries in a friend (light RRR = 2.13, moderate RRR = 3.42, heavy RRR=5.32), low parental monitoring—staying out late or overnight (late: light RRR = 1.62, moderate RRR = 2.44, heavy RRR = 2.32; overnight: light RRR = 1.57, moderate RRR = 1.94, heavy RRR = 3.38), health-damaging behaviors (per unit increase: light RRR = 1.91, moderate RRR = 3.15, heavy RRR = 5.03), and depressive symptoms (per scale point increase light RRR = 1.03, moderate RRR = 1.09, heavy RRR = 1.11). Confiding in a parent was associated with lower likelihood of intimate activity (light RRR = .82, moderate RRR = .65, heavy RRR = .65).

**Conclusions:**

Partnered intimate activity of some form is commonplace among 14-year-olds in the United Kingdom. Given the short- and long-term implications of adolescent sexual development and well-being, improving our understanding of influences could help identify opportunities for interventions with benefits across the lifecourse.

Partnered intimate activity in adolescence is increasingly considered a normative part of development [1,2], as experimentation and pushing boundaries around behaviors are viewed as being part of developing autonomy and identity. Although, it may be that this normative view of development and progressing into adulthood varies in different country contexts. Much of the prior work on intimate activity in adolescence has had a narrow focus on the timing and circumstances of sexual debut, usually referring specifically to first vaginal intercourse. Younger age at sexual debut correlates with unplanned teenage pregnancy, sexually transmitted infections, poor mental health, and lower educational attainment although the direction of association between these factors is not always clear cut [3–10].

Early sexual debut generally refers to having had sexual intercourse before the age of 16 years, with the most recent British prevalence estimates from two sources: the third British National Survey of Sexual Attitudes and Lifestyles, which reported that 30% of young adults born in the 1980s and 1990s engaged in sexual intercourse before the age of 16 years [11]; and the Avon Longitudinal Study of Parents and Children (ALSPAC) of young adults born at the beginning of the 1990s, which showed that 18% of 15-year-olds reported having had sexual intercourse [12]. Similarly, the U.S. Youth Risk Behavior Surveillance Survey reported that 15% of respondents had had sexual intercourse before the age of 15 years [13]. ALSPAC collected data in the mid-noughties on a broader range of intimate partnered activities among 12- to 13-year-olds reporting that 44% had held hands, 32% had kissed, and 5% had engaged in underclothes touching [14]. However, little is known about the patterning of a broader range of intimate practices among contemporary adolescents in the United Kingdom.

Most prior research examining patterns in emerging intimate and/or sexual activity and the factors associated with different patterns comes from settings other than the United Kingdom. A Dutch study found that three quarters of young people progressed through a sequence of activities from those classified by the researchers as “less intimate,” including hand-holding and kissing, through to “more intimate” activities, such as sexual intercourse [15]. Data on U.S. adolescents by Haydon et al. [16] identified five distinct patterns of initiating heterosexual behavior, the most common being first having vaginal intercourse with the initiation of other practices, such as oral-genital contact and anal intercourse, occurring at least 1 year later. However, Haydon et al.’s article did not look at these behaviors relative to the initiation and timing of nongenital sexual activities. Looking at a broader range of intimate activities is important, as there is most often a pathway of activities before sexual debut, providing potential opportunities to intervene, not to prevent young people from engaging in intimate activities as this is part of normal development but to do so safely to protect and promote their sexual well-being and their well-being more broadly.

Prior research suggests that various aspects of young people’s lives encourage or constrain different levels and types of intimate activity. For example, it is suggested that supportive family relationships are associated with lower rates of sexual debut in early adolescence [17–20]. On the other hand, hostile parenting, social disadvantage, and behaviors including binge drinking and substance use are associated with higher rates of sexual debut before age 16 years [12,16,20–22]. A recent study suggested that close communicative peer relations were associated with early sexual debut [23], and another study suggested that having peers as a main source of information about sex was associated with poor sexual outcomes [24]. Although considerable attention has been paid to the correlates of sexual debut in early adolescence, if intimate partnered activities are considered a normal part of development, it is also important to examine factors associated with noncoital activities. One Canadian study reported that supportive parenting was associated with “light” intimate activity [20]. To date, little is known about potential influences on emerging intimate and/or sexual behavior in early adolescence. Improving our understanding of the factors at play has important implications for sexual health and broader social consequences, thus informing policy and intervention programs [25], providing opportunities for young people to develop skills, for example, around negotiation and communication in relationships.

In this article, data from a large representative UK population–based survey, the Millennium Cohort Study on over 11,000 participants aged 14 years are used to investigate:

1. The prevalence of partnered intimate activities grouped into three categories: “light” (handholding, kissing, and cuddling); “moderate” (touching and fondling under clothes); and “heavy” (oral sex and sexual intercourse).
2. Associations between potential correlates including social relationships, parental monitoring and supervision, health behaviors, and psychosocial well-being with light, moderate, and heavy intimate activities.

Given that exploration of romantic and intimate experiences is a normal part of development, we expect that most study participants will have experience of partnered intimate activities. We hypothesize that conflictual family relationships, low levels of parental monitoring, confiding peer relationships, potentially health-damaging behaviors, and poor psychosocial well-being will be most strongly associated with heavier partnered intimate activities. This article is in the main descriptive, and we do not attempt to disentangle the relative importance of different aspects of young people’s lives in relation to their experiences of partnered intimate activities.

## Methods

The Millennium Cohort Study is the most comprehensive survey of adolescent health and development in the United Kingdom. It is a nationally representative prospective cohort study of children born into 19,244 families between September 2000 and January 2002 (http://www.cls.ioe.ac.uk/shared/get-file.ashx?id=1806&itemtype=document). Participating families were selected from a random sample of electoral wards with a stratified sampling design to ensure adequate representation of all four UK countries, disadvantaged and ethnically diverse areas. The first sweep of data was collected when cohort members were around 9 months, and the subsequent five sweeps of data were collected at ages 3, 5, 7, 11, and 14 years [26]. At the age 11 and 14 sweeps (corresponding to 2011–2012 and 2014–2015, respectively), cohort members and their carers were interviewed during home visits. At this time, cohort members self-completed computer-assisted questionnaires in private including items about social relationships and health behaviors, and in addition, at age 14 years, they answered questions about intimate partnered activity. Carers (96% of whom were cohort members’ parents and for ease throughout are referred to as parents) answered questions about socioeconomic circumstances and family relationships. Interview data were available for 61% of families when cohort members were aged 14 years.

At age 14 years, partnered intimate activity was assessed with a series of questions answered by cohort members about whether they had engaged in intimate activities (adapted from those used in the ALSPAC study [12]) with another young person in the last 12 months (henceforth “partnered intimate activity” for brevity).

Questionnaire items were asked in an order assuming progression from less to more intimate activities. For example, participants who said they had held hands or kissed or cuddled went on to be asked questions about touching under clothing and fondling. In turn, participants who said they had engaged in touching or fondling were asked about oral sex and sexual intercourse. We collapsed items to construct mutually exclusive “light,” “moderate,” and “heavy” categories as follows:

- “light” answered “yes” to any of the followingdheld hands with another or kissed on the mouth by/kissed another or cuddled with another;
- “moderate” answered “yes” to any of the followingdtouched by/touched another under clothes or fondled by/fondled another’s private parts;
- “heavy” answered “yes” to any of the followingdhad oral sex performed on/performed on another, had sexual intercourse.

These categories, although subjectively defined, do convey a social meaning in terms of progression of activitiesdintimacy over clothing, intimacy under clothing, and intimacy involving genital contact. Appendix Table 1 shows the percentages of each reported activity, and that the distribution of these activities was similar for boys and girls.

### Covariates

Questionnaire responses were used to capture numerous dimensions of cohort members lives: social relationships with friends and parents including having confiding and conflictual relationships; social support from friends and family; parental supervision and monitoring including staying out late and overnight; health-damaging behaviors including alcohol, cigarette, and substance use (binge drinking, cigarette smoking, and illegal drug use—a summed score was created to indicate engagement in any of these behaviors); and psychosocial well-being including self-esteem [27], level of educational engagement, social and emotional difficulties, and depressive symptoms [28]. Appendix Table 2 shows full details of items used and response categories.

For ease of use in bivariate descriptive analyses, we created binary indicator variables for self-esteem and depressive symptoms, but continuous variables were used in our multivariate models.

We used control variables in our analyses, which had previously been shown to correlate with adolescent intimate partnered activity as follows: gender [12], early puberty [20] (age 11 years, data on reported menarche in girls and facial hair growth or voice change or body hair in boys), family structure [21] (two vs. one parent), and equivalized household income quintiles [12]. Birth order, migrant generation, and the presence of residential grandparents were considered, but these factors were not independently associated with reported intimate activity and were therefore not included in analyses.

### Study sample

We analyzed data on cohort members for whom information on partnered intimate activity was available. The analytical sample was 11,079 after multiply imputing missing values on covariates due to item nonresponse, with the amount of missing variable data ranging from 0% to 14%. We used multiple imputation, which accounts for uncertainty about missing values by imputing several values for each missing data point [29]. We generated 25 filled-in data sets and report consolidated results from all imputations using Rubin’s combination rules [29] and excluded cases with imputed values on sexual activity from the analytical sample to improve the efficiency of estimates [30]. Results from the imputed analyses did not vary substantively from the analyses using listwise deletion (data not shown and available on request).

### Statistical analysis

To examine whether there were associations between potential correlates with partnered intimate activity, we ran multivariable multinomial regression models, with no reported intimate activity as the reference category. A fully adjusted model simultaneously adjusting for all variables is presented. In supplementary analyses, we included variables (where available) from earlier in adolescence when cohort members were aged 11 years to assess whether preceding markers of social relationships, parental supervision and monitoring, health behaviors, and psychosocial well-being were associated with intimate partnered activity at age 14 years (Appendix Table 3). All analyses were carried out using Stata version 15.1 (Stata Corp). Survey weights were applied throughout to take account of the unequal probability of being sampled and survey attrition.

Ethical approval was not required for this study, as the analysis involved secondary analysis of publicly available data.

## Results

### How common are partnered intimate activities?

Cohort members were on average 14.3 (standard deviation .34) years of age. Three of 10 study participants reported not having engaged in any form of partnered intimate activity. More than half (58 percent) reported “light” activity, 7.5% reported “moderate” activity, and 3.2% “heavy” activity. Boys were slightly more likely to report moderate activity compared with girls (8.7% vs. 6.3%, respectively), but there were no gender differences for light or heavy activity. Moderate and heavy activities were more common among cohort members for whom puberty had started before the age of 11 years (10.4% vs. 6.6% and 5.5% vs. 2.5%, respectively). There was a weak association with family socioeconomic position. Participants from one parent families were more likely to report engaging in intimate activity and specifically heavy activity (Table 1), but these differences were attenuated in multivariable analysis.

In a nonhierarchical manner, associations between contextual factors—social relationships, parental monitoring, health behaviors, and psychosocial well-being with partnered intimate activities—are described in the following sections. We first comment on bivariate results followed by multivariate findings. Finally, we comment on analyses that examined associations between contextual factors from earlier in the lifecourse—at age 11 years with partnered activities by age 14 years.

### Social relationships

As expected, in general, participants with close, confiding friendships were more likely to report intimate activity, whereas the opposite was apparent for having close, confiding, non-conflictual relationships with parents, which were associated with a lower likelihood of intimate activity (Table 1). In multivariable models, some, but not all, of these associations remained statistically significant. Young people who confided worries in a friend were more likely to report intimate activity (light relative risk ratio [RRR] = 2.13, moderate RRR = 3.42, heavy RRR = 5.32). Likewise, arguing with one’s parents most days was associated with intimate activity (light RRR = 1.57, moderate RRR = 2.35, heavy RRR = 2.05), while confiding in a parent was associated with lower likelihood of intimate activity (light RRR = .82, moderate RRR = .65, heavy RRR = .65). Weak social support was associated with the lower likelihood of intimate activity, the association for heavy activity losing statistical significance in the fully adjusted model (Table 2 light RRR = .73, moderate RRR = .68, heavy RRR = .93).

### Parental monitoring

Overall, lower levels of parental supervision and monitoring were associated with participants being more likely to report any intimate activity (Table 1). Cohort members who reported their parents not knowing their whereabouts were more likely to report intimate activity (Table 2; light RRR = 1.38, moderate RRR = 1.89, heavy RRR = 2.11). Similar patterns were apparent for cohort members who, without their parents knowing where they were, stayed out late (light RRR = 1.62, moderate RRR = 2.44, heavy RRR = 2.32), or overnight (light RRR = 1.57, moderate RRR = 1.94, heavy RRR = 3.38; Table 2).

### Health-damaging behaviors

Health-damaging behaviors were associated with all levels of intimate activity in the expected direction—per unit increase in health behavior score—light RRR = 1.91, moderate RRR = 3.15, heavy RRR = 5.03 (Table 2).

### Psychosocial well-being

High self-esteem scores were associated with lower rates of any intimate activity (Table 1). In contrast, in fully adjusted models, per unit increase on the self-esteem scale was associated with increased likelihood of intimate activity (light RRR = 1.02, moderate RRR = 1.07, heavy RRR = 1.04), although all estimates were in the same direction only the association with moderate activities was statistically significant. Higher levels of educational engagement were associated with lower prevalence of any intimate activity (Table 1), but associations lost statistical significance in the fully adjusted model. Cohort members with higher current depressive symptom scores were more likely to report any intimate activity (in multivariate models, for each scale point increase, Table 2 light RRR = 1.03, moderate RRR = 1.09, heavy RRR = 1.11).

### Contextual factors from early adolescence

Consistent with contemporaneous associations, markers of social relationships, supervision, health behaviors, and psychosocial well-being from earlier in adolescence were associated with the likelihood of intimate activity by age 14 years. For instance, at age 11 years, conflictual family relationships, low levels of parental supervision, and cigarette and alcohol use were all associated with higher rates of intimate activity and high levels of educational engagement with lower rates of intimate activity (Table 1). However, with the exception of low parental supervision, none of these associations remained statistically significant when age 14 years variables were taken into account (Appendix Table 3).

## Discussion

Most 14-year-olds reported having engaged in some form of intimate activity with another young person in the past year; for the majority, this was “light” activity with just one in 30 reporting “heavy” activity. Approximately three in 10 reported no experience of intimate activity with a partner. We observed similar patterns of reporting by gender. These findings underscore the fact that intimate partnered exploration is a normative part of adolescent development for both boys and girls. The wider literature has focused on potential pitfalls associated with sexual debut before the age of 16 years and has given less attention to what might be considered less intimate activities. In an attempt to provide a more comprehensive picture of young people’s lives, we examined different potential spheres of influence across a broad range of intimate activities, informing potential points for intervention, not to stop young people from engaging in intimate activities as this is part of normative development, but rather to mitigate potential risks and protect their sexual and broader well-being.

In line with hypothesized associations, our findings suggest that having close confiding friendships, low levels of parental monitoring, engaging in health behaviors such as drinking alcohol, smoking, and drug use, and having depressive symptoms were strongly associated with an increased likelihood of any intimate activity. This points to the fact that young people explore intimate behaviors and often push boundaries associated with other behaviors such as drinking and smoking in tandem with one another. We also found that close, confiding nonconflictual parental relationships were associated with a reduced likelihood of intimate activity.

In general, there was a suggestion that correlates were associated in a stepwise fashion with an increased magnitude of association from light through to heavy activity; however, overlapping confidence intervals for estimates indicate weak evidence in support of this. We observed that common contextual factors from earlier in the lifecourse were associated with intimate activities by age 14 years hinting at continuities in social relations, parental monitoring, and supervision for example.

Our finding that most 14-year-olds reported engaging in some form of partnered intimate activity is consistent with British National Survey findings, although Natsal-3 asked participants specifically about their age at first sexual experience (subjectively defined) with someone of the opposite sex, the median age being 14 years [31]. Unlike findings from ALSPAC [12], we found no strong evidence of gender differences in reported intimate activities. In common with a U.S. study [21], we found that young people from one parent families were more likely to report heavy intimate activity, although this association did not persist when contextual factors including relationships with family and friends were taken into account. We did not find, as has been shown elsewhere [12,16], that other markers of socioeconomic disadvantage were linked to intimate activity. In common with other studies [17–20], we found that supportive family relationships were linked to lower rates of heavy intimate activity. Consistent with findings from a large meta-analysis [22], we found that intimate activity was associated with alcohol and substance use. Taken together, our findings highlight the importance of initiatives aiming to minimize risk and promote well-being in youth and the need to look holistically at intimate activities, health behaviors, and social relationships.

Our study has distinct strengths. First, we used data from a large-scale representative contemporary UK setting, making our findings generalizable to the wider population. Second, we were able to simultaneously investigate multiple dimensions of young people’s lives in relation to their engagement in partnered intimate activities. Third, we were able to investigate associations across a range of partnered intimate activities rather than just focusing on sexual intercourse, thus potentially more usefully informing policy and sex education programs. On the other hand, our study has limitations; for instance, data were collected in a way which assumed that the initiation of intimate activity follows a linear path from holding hands and cuddling through to oral sex and intercourse. However, findings from elsewhere suggest that significant proportions of young people do not follow so-called “linear” patterns of partnered intimate experiences [15,16]; thus, our study is likely to have underestimated the prevalence of “heavier” sexual activities including oral sex and intercourse. This data limitation makes it impossible to identify arguably the most vulnerable young people whose first partnered intimate experience involves oral sex or intercourse without having first experienced what might be considered less intimate activities. Available data did not cover the entire repertoire of intimate activities; for instance, no information was available about solo masturbation or anal intercourse. Furthermore, the questions used asked about activities with other young people, thus would not capture child sexual abuse. Data were self-reported and thus prone to some reporting bias, and there could be some gender differences in this with boys potentially more likely than girls to exaggerate and/or girls being less likely to report. The gender of young people with whom study participants had experiences was not known; therefore, it was not possible to investigate intimate activities with people of the same sex. Finally, the data are cross-sectional, and causal inference cannot be drawn from our findings, although we did take account of social relationships and health behaviors from earlier in adolescence in our analyses.

Our finding that most 14-year-olds reported some form of intimate activity with another young person in the past year highlights the importance of timely and comprehensive sex and relationships education to support young people to navigate positive intimate and/or sexual experiences—that is, those that are mutually wanted, protected, and pleasurable. The concept of “sexual competence”—used to refer to sexual experiences characterized by autonomy, an equal willingness of partners, being “ready” and (when relevant) protected by contraceptives—is important at all ages. In our study, one in thirty 14-year-olds had had oral sex and/or sexual intercourse; however prior research suggests that only one fifth of girls and a third of boys having sex at this age are sexually competent [32]. Furthermore, a lack of competence at first vaginal intercourse has been associated with later poor sexual health [33]. Our finding that sexual activities tended to cluster with potentially health-damaging behaviors including binge drinking, smoking, and drug use, both contemporaneously and from earlier in adolescence, underlines the importance of holistic interventions and supports the implementation of broad health promotion programs earlier in childhood. The family setting is also relevant; our findings suggesting that family context is potentially important in shaping young people’s intimate experiences, and other studies highlight the importance for young people of timely information about sex from parents [24].

Partnered intimate activity of some form, although typically “light” activity, is commonplace among 14-year-olds living in the United Kingdom, and multiple factors related to family context, friendships, health behaviors, and mental health are associated with a broad range of intimate activities. Given the short- and long-term implications of the development of adolescent intimate and/or sexual experiences and well-being, improving our understanding of influences could help identify opportunities for interventions with benefits across the lifecourse.

## Supplementary Material

Supplementary data related to this article can be found at https://doi.org/10.1016/j.jadohealth.2019.04.028.

Appendix table 2

Appendix table 3

Appendix table 1

## Figures and Tables

**Table 1:** Prevalence (%) of partnered intimate activities by social relationships, parental supervision, health behaviors, psychosocial well-being, and controls (n = 11,079)

	No sexual activities (n = 3,707)	Light (n = 6,298)	Moderate (n = 744)	Heavy (n = 330)
Overall prevalence (%)	31.8	57.5	7.5	3.2
Gender				
Boys	31.0	57.0	8.7	3.2
Girls	32.6	58.0	6.3Note *	3.1
Social relations and support				
Cohort member report				
Argues with friends (age 11 y)				
Less often than once a month/never/no friends	33.2*	56.8	7.2	2.7*
At least once a month	29.1	58.9	8.1	3.9
Most days/at least once a week (ref)	28.9	58.8	8.1	4.2
Has close friends				
Yes	31.2*	58.0*	7.6*	3.2
No	52.4	40.7	4.7	2.2
Closeness to mother
Extremely/very close	33.3	57.6	6.8	2.2
Not close/no mother	25.3*	57.1	10.4*	7.3*
Closeness to father				
Extremely/very close	34.4	56.9	6.6	2.0
Not close/no father	27.1*	58.5	9.1*	5.2*
Argues with parents				
Hardly ever (ref)	38.4	55.2	5.0	1.4
Less than once per week	31.3*	58.4*	7.6*	2.7*
Once or more per week	24.9*	61.2*	9.1*	4.8*
Most days	19.7*	57.9	14.4*	7.9*
Not applicable	45.7*	49.2*	3.1*	2.0
Confides in parents
No	25.9	59.1	10.1	5.0
Yes	37.2*	56.1*	5.2*	1.5*
Confides in a friend				
No	38.8	53.5	5.6	2.1
Yes	19.9*	64.3*	10.7*	5.1*
Social support
Strong	31.9	58.2*	7.3*	2.7*
Weak	30.9	53.2	9.2	6.6
Parent report				
Frequent battles with child (age 11 y)				
No	33.2	57.3	6.8	2.8
Yes	29.4*	58.0	8.7*	3.9*
Closeness to child				
Extremely/very close	32.2*	57.9	7.2*	2.8*
Not very/fairly close	27.7	53.6	10.7	8.0
Parent quarrels with child				
Hardly ever (ref)	37.0	55.2	5.7	2.1
Less than once per week	30.0*	59.4*	7.8*	2.8
Once or more per week	29.3*	58.1	8.7*	3.9*
Most days	30.1*	55.7	8.5*	5.7*
Parental supervision and monitoring				
Cohort member report				
Unsupervised time with friends (age 11 y)				
Rarely (ref)	41.9	51.0	5.1	2.0
Sometimes	30.7*	59.3*	7.5*	2.6
Often	24.2*	61.4*	9.6*	4.8*
Parents know child’s whereabouts
Always/usually	35.9	56.9	5.5	1.7
Sometimes/never	16.2*	59.9*	15.1*	8.7*
Stay out after 9 p.m.				
No	36.6	56.6	5.2	1.6
Yes	14.3*	60.9*	15.9*	8.9*
Stay out overnight				
No	33.3	57.7	6.8	2.2
Yes	12.3*	55.4	17.0*	15.4*
Parent report				
Unsupervised time (age 11 y)				
Rarely (ref)	39.7	53.5	4.8	1.9
Sometimes	29.9*	58.6*	8.3*	3.2*
Often	26.1*	60.3*	9.3*	4.3*
Health behaviors, cohort member report				
Any cigarette or alcohol use (age 11 y)				
No	33.6	57.0	6.6	2.7
Yes	20.5*	60.5*	13.1*	5.9*
Any cigarette, e-cigarette, binge drinking, illicit drug use				
No	47.3	50.1	2.2	0.3
Yes	18.2*	64.0*	12.2*	5.7*
Psychosocial well-being, cohort member report				
Self-esteem (age 11 y)				
High	30.9	59.6	7.2	2.4
Not high	31.9	57.2	7.6	3.3
Self-esteem				
High	36.0*	54.6*	7.0	2.3
Not high	31.1	58.0	7.6	3.3
Educational engagement (age 11 y)				
high	40.7*	52.0*	5.9*	1.3*
Not high	30.6	58.3	7.7	3.4
Educational engagement				
High	46.2*	50.1*	3.1*	.6*
Not high	30.1	58.4	8.0	3.5
Clinically relevant depressive symptoms				
No	35.1*	56.9	6.2*	1.8*
Yes	23.6	59.0	10.9	6.6
Control variables at age 11 y				
Puberty				
No	33.2*	57.7	6.6*	2.5*
Yes	27.1	57.0	10.4	5.5
Family structure				
Two parent family	33.0*	57.1	7.2	2.7*
One parent family	27.1	59.3	8.6	5.0
Family income, quintiles				
Poorest	35.1	54.7	6.4	3.8
Second	31.4	56.8	7.2	4.5*
Third	29.6	59.0	8.0	3.3
Fourth	32.6	57.7	7.5	2.1
Richest (ref)	31.4	58.0	7.7	2.9

Prevalence estimates are weighted with Millennium Cohort Study sample weights. Sample size is not weighted. Significance testing is within sexual activity group, *p < .05.

**Table 2:** Relative risk ratios (95% confidence intervals) from multivariable multinomial regressions, associations of covariates with partnered intimate activity, N = 11,079

	Ref: no sexual activities	Light	Moderate	Heavy
Social relations and support				
No close friends		.53*** (.38–.74)	.51 (.25–1.03)	.33 (.09–1.21)
Closeness to mother				
Extremely/very close (ref)				
Not close/no mother		.99 (.86–1.14)	.93 (.70–1.24)	1.17 (.78–1.74)
Closeness to father				
Extremely/very close (ref)				
Not close/no father		1.02 (.89–1.16)	.98 (.77–1.24)	1 (.70–1.41)
Argues with parents				
Hardly ever (ref)				
Less than once per week		1.09 (.96–1.24)	1.16 (.91–1.48)	1.18 (.78–1.79)
Once or more per week		1.27** (1.07–1.51)	1.35 (.99–1.84)	1.68* (1.04–2.72)
Most days		1.57*** (1.26–1.94)	2.35*** (1.65–3.36)	2.05** (1.22–3.44)
Not applicable		.93 (.74–1.16)	.65 (.35–1.22)	1.41 (.59–3.38)
Confides in …				
Parents		.82** (.74–.93)	.65*** (.51–.83)	.65* (.44–.96)
A friend		2.13*** (1.88–2.40)	3.42*** (2.74–4.26)	5.32*** (3.89–7.26)
Weak social support		.73*** (.61–.87)	.67* (.50–.91)	.93 (.64–1.34)
Parent is not close to child		.92 (.74–1.13)	1.14 (.82–1.60)	1.51 (.94–2.42)
Parent quarrels with child				
Hardly ever (ref)				
Less than once per week		1.18* (1.02–1.37)	1.29 (.99–1.67)	1.14 (.73–1.80)
Once or more per week		1.09 (.93–1.28)	1.23 (.92–1.64)	1.35 (.84–2.15)
Most days		.91 (.71–1.17)	.75 (.52–1.09)	.89 (.46–1.72)
Parental supervision and monitoring				
Parents do not know child’s whereabouts		1.38*** (1.17–1.63)	1.89*** (1.44–2.48)	2.11*** (1.50–2.96)
Stay out after 9 p.m.		1.62*** (1.38–1.89)	2.44*** (1.90–3.13)	2.32*** (1.65–3.28)
Stay out overnight		1.57** (1.19–2.06)	1.94*** (1.33–2.84)	3.38*** (2.24–5.12)
Health behaviors				
Health behavior score		1.91*** (1.75–2.08)	3.15*** (2.79–3.56)	5.03*** (4.24–5.95)
Psychosocial well-being				
Self-esteem score		1.02 (1.00–1.05)	1.07** (1.02–1.12)	1.04 (.97–1.11)
Educational engagement		.99 (.96–1.02)	.99 (.94–1.04)	1.03 (.95–1.11)
Depressive symptoms score		1.03*** (1.02–1.04)	1.09*** (1.06–1.11)	1.11*** (1.08–1.15)
Control variables				
Puberty		1.15 (1.00–1.32)	1.51** (1.17–1.93)	2.14*** (1.49–3.08)
One parent family		1.14 (.99–1.31)	1.07 (.83–1.39)	1.17 (.81–1.70)
Family income, quintiles (richest is ref)				
Poorest		.75** (.61–.92)	.44*** (.30–.66)	.43** (.25–.74)
Second		.86 (.72–1.02)	.63** (.44–.88)	.73 (.44–1.21)
Third		.92 (.77–1.10)	.75 (.55–1.01)	.61 (.36–1.01)
Fourth		.91 (.78–1.05)	.82 (.60–1.11)	.54* (.33–.87)
Child is girl		.93 (.81–1.06)	.59*** (.47–.73)	.62* (.41–.94)
Child’s age at interview (y)		1.00 (.86–1.17)	1.54** (1.14–2.07)	2.60*** (1.66–4.09)

All estimates are weighted with MCS sample weights. ***p < .001, **p < .01, *p < .05.
