# Supplementary material for: Partnered Intimate Activities in Early Adolescence—Findings From the UK Millennium Cohort Study
Source: J Adolesc Health. Author manuscript; Available in PMC 2020 Jan 28. (PMC6986910; doi:10.1016/j.jadohealth.2019.04.028)
Supplement: Appendix table 2 [file EMS85439-supplement-Appendix_table_2.docx]

**Appendix Table 2**

*Social relationships and social support were assessed by cohort member and parent responses (at the age 14 sweep unless otherwise noted) to questions as follows.*

Cohort member reported:

- argues with friends (age 11 - less than monthly/never, at least once per month, most days/at least once per week);
- has close friends (yes vs no);
- closeness to mother and father (extremely/very, not very/fairly, no parent/not in contact);
- argues with parents (hardly ever, less than once per week, once or more per week, most days, not applicable);
- confiding relationship with a parent, a friend (yes vs no);
- social support – we generated a dichotomised variable (strong vs weak), derived from a sum scale of 3 items (has family/friends with whom feel safe, secure, happy; has someone to trust when have problems; feels close to someone).

Parent reported:

- frequent battles with child (age 11 - yes vs no);
- closeness to child (extremely/very vs not very/fairly);

**-** parent quarrels with child (hardly ever, less than once per week, once or more per week, most days).

*Parental supervision and monitoring were assessed from cohort member and parent responses (from age 14 sweep unless otherwise noted) to questions as follows.*

Cohort member reported:

- unsupervised time with friends after school and at weekends (age 11 - rarely, sometimes, often);
- parents know whereabouts (always/usually vs sometimes/never);
- stayed out after 9 pm (in the last 12 months) without parent knowing where (yes vs no);
- stayed out overnight (in the last 12 months) without parent knowing where (yes vs no).

Parent reported:

- unsupervised time after school and at weekends (age 11 - rarely, sometimes, often).

*Health behaviours*

Cohort members answered questions about cigarette, alcohol and other drug use and summed scores from binary indicators were created as follows: age 11 - ever smoked a cigarette and ever had an alcoholic drink (range 0-2); and at age 14 current (last 12 months) cigarette and e-cigarette use, binge drinking (5 or more drinks on a single occasion), and illicit drug use (range 0-4).

*Psychosocial wellbeing*

Cohort members’ reports were used to assess aspects of wellbeing as follows:

self-esteem (ages 11 and 14 was assessed using the Rosenberg scale from which we created a dichotomised variable (high vs not high) derived from the sum of items on self-satisfaction: having good qualities; able to do things similar to others; person of value; and feel good about oneself);

educational engagement (ages 11 and 14 was assessed by summing responses to the following: how often try your best, find school interesting, feel unhappy at school, get tired at school, feel school is a waste of time);

*Depressive symptoms*: participants completed the Mood and Feelings Questionnaire – short version (SMFQ) from which a summed score was created. The SMFQ comprises 13 items on affective symptoms in the last 2 weeks as follows: felt miserable or unhappy; didn’t enjoy anything at all; so tired just sat around and did nothing; was very restless; felt I was no good anymore; cried a lot; found it hard to think properly or concentrate; hated myself; was a bad person; felt lonely; thought nobody really loved me; thought I could never be as good as other kids; did everything wrong.
