# Supplementary material for: Partnered Intimate Activities in Early Adolescence—Findings From the UK Millennium Cohort Study
Source: J Adolesc Health. Author manuscript; Available in PMC 2020 Jan 28. (PMC6986910; doi:10.1016/j.jadohealth.2019.04.028)
Supplement: Appendix table 3 [file EMS85439-supplement-Appendix_table_3.docx]

| **Appendix Table 3. Relative risk ratios from multinomial regressions, associations of indicators from earlier in adolescence with partnered intimate activity, N = 11,079** | | | | | | | | | |
| --- | --- | --- | --- | --- | --- | --- | --- | --- | --- |
|  |  | **Age 11 variables only** | | |  | **Ages 11 and 14 variables** | | | |
|  | Ref: No sexual activities | Light | Moderate | Heavy |  | Light | Moderate | Heavy | |
| **Social relations and support** |  |  |  |  |  |  |  |  | |
| Argues with friends |  |  |  |  |  |  |  |  | |
| Less often than once a month/Never/No friends |  | 0.93 (0.79 - 1.09) | 0.92 (0.68 - 1.25) | 0.82 (0.54 - 1.26) |  | 0.97 (0.81 - 1.16) | 1.04 (0.74 - 1.47) | 0.96 (0.57 - 1.64) | |
| At least once a month |  | 1.02 (0.86 - 1.22) | 1.04 (0.74 - 1.46) | 1.1 (0.69 - 1.76) |  | 1.03 (0.84 - 1.26) | 1.13 (0.78 - 1.64) | 1.25 (0.71 - 2.18) | |
| Most days/at least once a week (ref) |  |  |  |  |  |  |  |  | |
| Frequent battles with child (parent report) |  | 1.09 (0.98 - 1.21) | 1.32^*^ (1.06 - 1.65) | 1.41^*^ (1.07 - 1.87) |  | 0.94 (0.83 - 1.06) | 0.95 (0.74 - 1.23) | 0.81 (0.56 - 1.16) | |
| **Parental supervision and monitoring** |  |  |  |  |  |  |  |  | |
| Unsupervised time with friends (CM report) |  |  |  |  |  |  |  |  | |
| Often |  | 1.80^***^ (1.55 - 2.09) | 2.19^***^ (1.64 - 2.91) | 2.71^***^ (1.75 - 4.19) |  | 1.48^***^ (1.27 - 1.74) | 1.39^*^ (1.01 - 1.92) | 1.48 (0.90 - 2.45) | |
| Sometimes |  | 1.45^***^ (1.28 - 1.64) | 1.58^***^ (1.23 - 2.02) | 1.38 (0.95 - 2.02) |  | 1.27^***^ (1.12 - 1.45) | 1.22 (0.93 - 1.60) | 1.03 (0.67 - 1.59) | |
| Rarely (ref) |  |  |  |  |  |  |  |  | |
| Unsupervised time (Parent report) |  |  |  |  |  |  |  |  | |
| Often |  | 1.22^*^ (1.05 - 1.42) | 1.71^***^ (1.29 - 2.26) | 1.55^*^ (1.02 - 2.36) |  | 1.09 (0.93 - 1.28) | 1.35 (0.99 - 1.85) | 0.99 (0.61 - 1.60) | |
| Sometimes |  | 1.22^**^ (1.07 - 1.39) | 1.73^***^ (1.31 - 2.29) | 1.57^*^ (1.04 - 2.37) |  | 1.14 (1.00 - 1.31) | 1.48^*^ (1.09 - 2.00) | 1.08 (0.66 - 1.75) | |
| Rarely (ref) |  |  |  |  |  |  |  |  | |
| **Health behaviours, CM report** |  |  |  |  |  |  |  |  | |
| Health behaviour score |  | 1.48^***^ (1.27 - 1.73) | 2.43^***^ (1.94 - 3.03) | 2.31^***^ (1.72 - 3.11) |  | 1.07 (0.89 - 1.28) | 1.27 (0.97 - 1.65) | 0.96 (0.69 - 1.33) | |
| **Psychosocial wellbeing, CM report** |  |  |  |  |  |  |  |  | |
| Self-esteem score |  | 1.02 (1.00 - 1.05) | 1.01 (0.97 - 1.06) | 0.98 (0.92 - 1.04) |  | 1.03^*^ (1.00 - 1.06) | 1.04 (0.99 - 1.10) | 1.03 (0.95 - 1.12) | |
| Educational engagement |  | 0.96^**^ (0.94 - 0.99) | 0.94^**^ (0.90 - 0.98) | 0.90^***^ (0.85 - 0.95) |  | 0.99 (0.97 - 1.03) | 1.02 (0.96 - 1.07) | 0.98 (0.91 - 1.05) | |
|  |  |  |  |  |  |  |  |  | |
| **Control variables at age 11** |  |  |  |  |  |  |  |  | |
| Puberty |  | 1.19^**^ (1.04 - 1.37) | 1.65^***^ (1.32 - 2.06) | 2.45^***^ (1.79 - 3.37) |  | 1.15 (1.00 - 1.32) | 1.51^**^ (1.17 - 1.93) | 2.14^***^ (1.49 - 3.08) | |
| One parent family |  | 1.27^***^ (1.11 - 1.44) | 1.45^**^ (1.16 - 1.82) | 1.90^***^ (1.41 - 2.55) |  | 1.14 (0.99 - 1.31) | 1.07 (0.83 - 1.39) | 1.17 (0.81 - 1.70) | |
| Family income, quintiles (richest is ref) |  |  |  |  |  |  |  |  | |
| Poorest |  | 0.70^***^ (0.57 - 0.84) | 0.49^***^ (0.35 - 0.70) | 0.61^*^ (0.39 - 0.97) |  | 0.75^**^ (0.61 - 0.92) | 0.44^***^ (0.30 - 0.66) | 0.43^**^ (0.25 - 0.74) | |
| Second |  | 0.82^**^ (0.70 - 0.95) | 0.66^**^ (0.48 - 0.89) | 0.89 (0.58 - 1.39) |  | 0.86 (0.72 - 1.02) | 0.63^**^ (0.44 - 0.88) | 0.73 (0.44 - 1.21) | |
| Third |  | 0.95 (0.81 - 1.13) | 0.87 (0.66 - 1.15) | 0.88 (0.58 - 1.34) |  | 0.92 (0.77 - 1.10) | 0.75 (0.55 - 1.01) | 0.61 (0.36 - 1.01) | |
| Fourth |  | 0.91 (0.79 - 1.04) | 0.83 (0.64 - 1.08) | 0.60^*^ (0.39 - 0.94) |  | 0.91 (0.78 - 1.05) | 0.82 (0.60 - 1.11) | 0.54^*^ (0.33 - 0.87) | |
| Child is girl |  | 1.12^*^ (1.00 - 1.24) | 0.94 (0.78 - 1.14) | 1.45^*^ (1.07 - 1.96) |  | 0.93 (0.81 - 1.06) | 0.59^***^ (0.47 - 0.73) | 0.62^*^ (0.41 - 0.94) | |
| Child's age at interview (years) |  | 1.26^**^ (1.08 - 1.46) | 2.32^***^ (1.79 - 3.02) | 4.03^***^ (2.72 - 5.97) |  | 1.00 (0.86 - 1.17) | 1.54^**^ (1.14 - 2.07) | 2.60^***^ (1.66 - 4.09) | |
| Notes: All estimates are weighted with MCS sample weights. CM-Cohort member. ^***^ p<0.001, ^**^ p<0.01, ^*^ p<0.05 | | | | | | | | |  |
