# Supplementary material for: Partnered Intimate Activities in Early Adolescence—Findings From the UK Millennium Cohort Study
Source: J Adolesc Health. Author manuscript; Available in PMC 2020 Jan 28. (PMC6986910; doi:10.1016/j.jadohealth.2019.04.028)
Supplement: Appendix table 1 [file EMS85439-supplement-Appendix_table_1.docx]

| **Appendix Table 1. Prevalence of partnered intimate activities by gender, n=11079** | | |
| --- | --- | --- |
|  | Boys | Girls |
| Held hands with a young person | 58.9 | 60.1 |
| Kissed or been kissed by a young person | 42.5 | 38.2 |
| Cuddled with a young person | 57.5 | 53.9 |
| Been touched under clothing by a young person | 11.0 | 8.8 |
| Touched a young person under clothing | 10.9 | 6.7 |
| Fondled a young person's private parts | 6.2 | 4.4 |
| Been fondled by a young person | 6.2 | 5.0 |
| Given or received oral sex | 3.2 | 2.9 |
| Sexual intercourse | 2.0 | 2.2 |
| Note: Prevalence estimates are weighted by attrition weights. | | |
